# GSTM1 and GSTT1 polymorphisms and nasopharyngeal cancer risk: an evidence-based meta-analysis

**DOI:** 10.1186/1756-9966-28-46

**Published:** 2009-04-01

**Authors:** Xianlu Zhuo, Lei Cai, Zhaolan Xiang, Qi Li, Xueyuan Zhang

**Affiliations:** 1 Department of Otolaryngology, Southwest Hospital, Third Military Medical University, Chongqing, PR China; 2 Institute of Hepatobiliary Surgery, Southwest Hospital, Third Military Medical University, Chongqing, PR China; 3 Guiyang Medical College, Guiyang, Guizhou Province, PR China

## Abstract

**Background:**

Previous evidence implicates polymorphisms of GSTM1 and GSTT1, candidates of phase II enzymes, as risk factors for various cancers. A number of studies have conducted on the association of GSTM1 and GSTT1 polymorphism with susceptibility to nasopharyngeal carcinoma (NPC). However, inconsistent and inconclusive results have been obtained. In the present study, we aimed to assess the possible associations of NPC risk with GSTM1 and GSTM1 null genotype, respectively.

**Methods:**

The associated literature was acquired through deliberate searching and selected based on the established inclusion criteria for publications, then the extracted data were further analyzed using systematic meta-analyses.

**Results:**

A total of 85 articles were identified, of which eight case-control studies concerning NPC were selected. The results showed that the overall OR was 1.42 (95%CI = 1.21–1.66) for GSTM1 polymorphism. While for GSTT1 polymorphism, the overall OR was 1.12 (95% CI = 0.93–1.34).

**Conclusion:**

The data were proven stable via sensitivity analyses. The results suggest GSTM1 deletion as a risk factor for NPC and failed to suggest a marked correlation of GSTT1 polymorphisms with NPC risk.

## Backgrounds

Nasopharyngeal cancer (NPC), a fast-growing tumour, characterized by a high frequency of nodal and distant metastasis at diagnosis, is rare in many areas of the world but common in Southeast Asia [1]. Evidence suggests that Epstein-Barr virus (EBV) infection is a major risk factor contributing to its tumorigenesis [2]. Besides, cigarette smoking and alcohol consumption are probably important etiological factors increasing the risk of developing NPC [3]. Moreover, environmental chemical pollutions, widely spread carcinogens, are difficult to be degraded in the environment and thus may have a long-term effect on human health. Despite many individuals exposed to EBV infection, environmental risk factors and/or with extensive tobacco and alcohol consumption, NPC develops only in a small group of exposed people, which suggests that genetic host factors might contribute to the carcinogenic mechanisms. Recent evidence indicates that carcinogen-metabolizing genes and DNA-repair genes may play critical roles in determining individual susceptibility to cancers. Polymorphisms in these genes encoding the enzymes, possibly by altering their expression and function, may increase or decrease carcinogen activation/detoxication and modulate DNA repair.

Xenobiotics can be detoxified by phase II enzymes, such as GSTM1 and GSTT1 which have been suggested to be involved in detoxification of polycyclic aromatic hydrocarbons (PAHs) and benzo(a)pyrene [4]. Evidence suggests that genetic polymorphisms of these genes might increase individual susceptibility to NPC. Therefore, a number of published studies have focused on GSTM1 and GSTT1 genetic variation with respect to NPC and have yielded conflicting results. Whether GSTM1 or GSTT1 polymorphism is a risk factor for NPC remains largely uncertain.

Since a single study may have been underpowered to clarify the associations of GSTM1 or GSTT1 polymorphisms with NPC susceptibility, in the present study we aimed to perform evidence-based quantitative meta-analyses that might increase statistical power to address this controversy.

## Methods

### Literature search strategy for identification of the studies

We carried out a search in the Medline, EMBASE and Chinese National Knowledge Infrastructure (CNKI) without a language limitation, covering all papers published up to Sep 2008, with a combination of the following keywords: *GSTM1*, *GSTT1*, *nasopharynx *or nasopharyngeal, *head and neck*, *cancer*, *carcinoma*, *tumour *or *neoplasm*.

We evaluated potentially associated publications by checking their titles and abstracts and then procured the most relevant publications for a closer examination. Moreover, the reference lists of the selected papers were also screened for other potential articles that possibly have been missed in the initial search. The following criteria were used for the literature selection of the meta-analysis:

1. Articles clearly describing studies in the association of NPC with GSTM1 or GSTT1 polymorphisms;

2. Case–control studies;

3. The NPC diagnoses and the sources of cases and controls should be stated;

4. The size of the sample, odds ratios (ORs) and their 95% confidence intervals (CIs) or the information that can help infer the results should also be offered;

5. Those publications that presented data allowing such outcomes to be derived were also selected.

Accordingly, the following exclusion criteria were also used:

1. Design and the definition of the experiments were obviously different from those of the selected papers;

2. The source of cases and controls and other essential information was not offered;

3. Reviews and repeated literature.

After searching, we reviewed all papers in accordance with the criteria defined above for further analysis. In addition, Hardy-Weinberg equilibrium test [5] was conducted to evaluate the genetic equilibrium for each study.

### Data extraction

Data were extracted and entered into a database. The extraction was performed by two reviewers independently. For conflicting evaluations, an agreement was reached following a discussion.

### Statistical analysis

The odds ratio (OR) of GSTM1 or GSTT1 polymorphisms and NPC risk was estimated for each study. For detection of any possible sample size biases, the OR and its 95% confidence interval (CI) to each study was plotted against the number of participants respectively. A Chi-square based Q statistic test was performed to assess heterogeneity. If the result of the heterogeneity test was P > 0.05, ORs were pooled according to the fixed-effect model (Mantel-Haenszel), Otherwise, the random-effect model (DerSimonian and laird) was used. The significance of the pooled ORs was determined by Z-test. The Hardy-Weinberg equilibrium was assessed via Fisher's exact test.

Publication bias was assessed by fail-safe number for P = 0.05 (Nfs0.05) [6]. Statistical analysis was undertaken using the program Review Manager 4.2 and SAS 8.1 software.

## Results

### Literature search and meta-analysis databases

**Figure 1:**
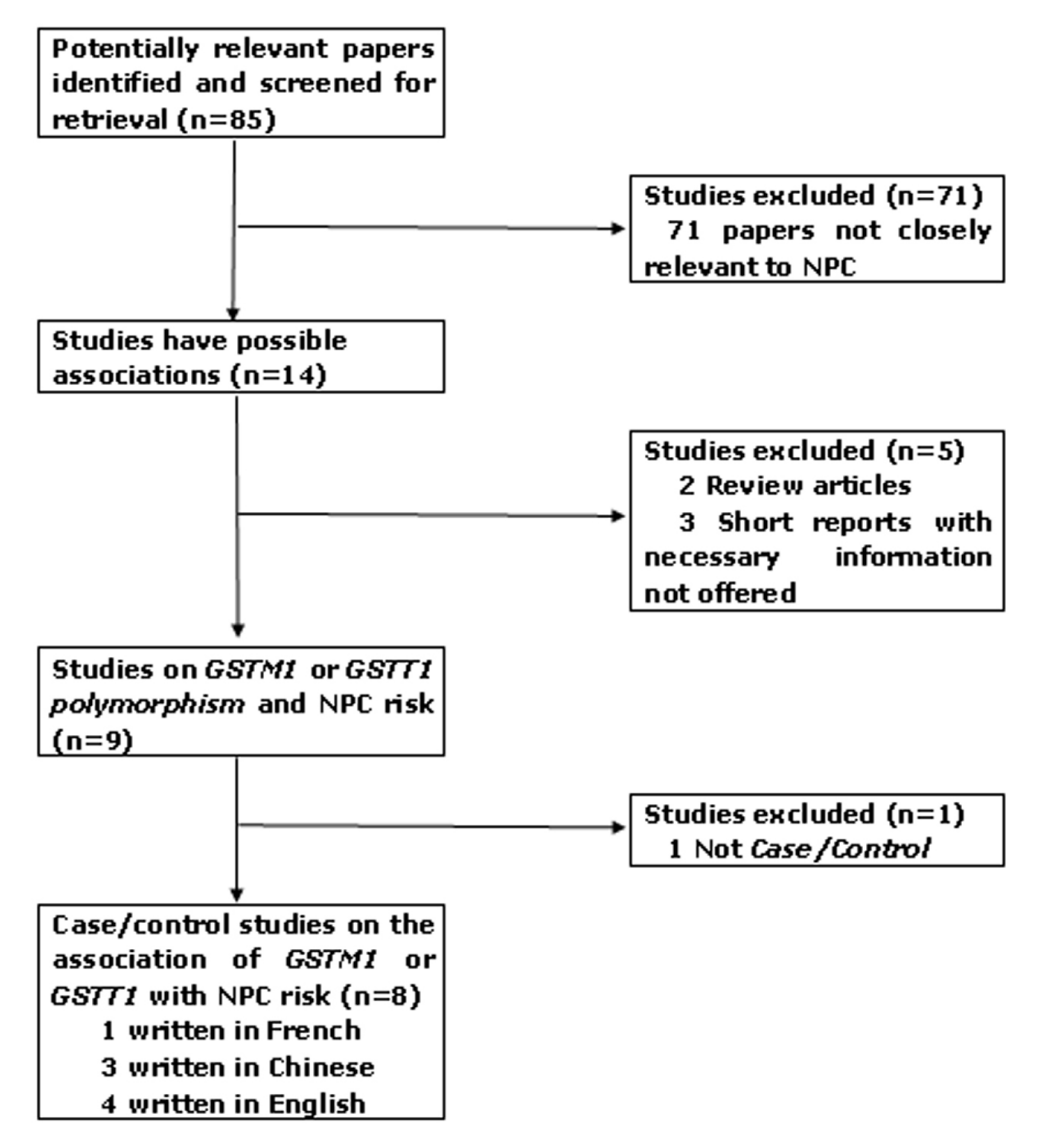
**The flow diagram of included/excluded studies**.

A total of 85 studies regarding GSTM1 or GSTT1 were identified (Fig. 1). After a careful review, irrelevant 71 papers were excluded. Then, the remaining 14 papers were examined according to our inclusion criteria and finally 4 concerning GSTM1 [7-10], and 4 concerning both of GSTM1 and GSTT1 [11-14] were selected. Of the included 8 studies, one was written in French [13], three in Chinese [8,9,12] and the remaining four studies [7,10,11,14] were written in English. The controls of the included studies are in agreement with Hardy-Weinberg equilibrium. We established a database according to the extracted information from each article. The information was listed in Tab. 1. According to the lists, the first author and the number of cases and controls for each study as well as other necessary information were presented.

**Table 1:** Case-control studies on GSTM1/GSTT1 polymorphisms and NPC risk

First Author	Publication Year	Cases	Controls	Histology	Ethnicity	genotype	Ref. number
Nazar-Stewart V	1999	83	142	11 Epithelial, Nos; 24 Undifferentiated; 48 Squamous	57 Caucasian; 7 African-American; 17 Asian; 2 Native American	GSTM1	[7]
Da SJ	2002	80	80	72 Squamous, 8 Adenocarcinoma	80 Asian (China)	GSTM1	[8]
Cheng YJ	2003	314	337	Not Determined	314 Asian (China)	GSTM1; GSTT1	[11]
Deng ZL	2004	91	135	91 Squamous	91 Asian (China)	GSTM1; GSTT1	[12]
Liao ZL	2005	80	72	Not Determined	80 Asian (China)	GSTM1	[9]
Tiwawech D	2005	78	145	Not Determined	78 Asian (Thailand)	GSTM1	[10]
Bendjemana K	2006	45	100	Not Determined	45 Caucasian (France)	GSTM1; GSTT1	[13]

Guo X	2008	341	590	Not Determined	341 Asian (China)	GSTM1; GSTT1	[14]

### Test of heterogeneity

**Figure 2:**
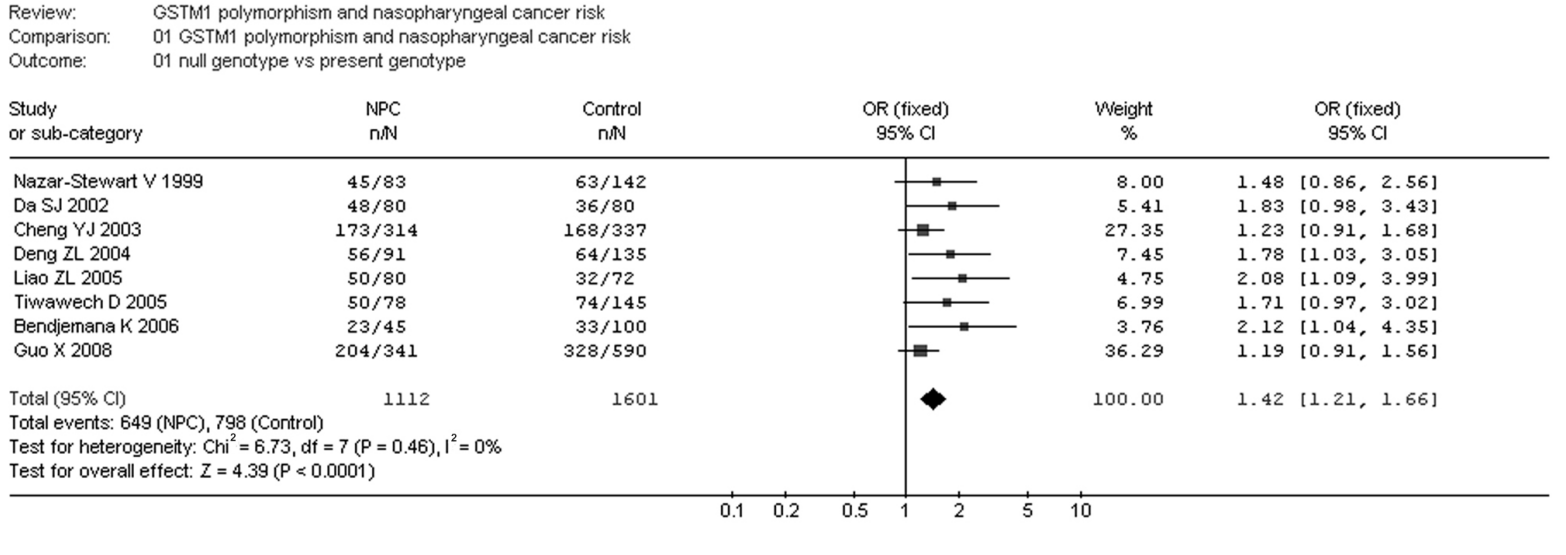
**Meta-analysis with a fixed-effect model for the association of NPC risk with GSTM1 polymorphism (null genotype versus present genotype)**.

**Figure 3:**
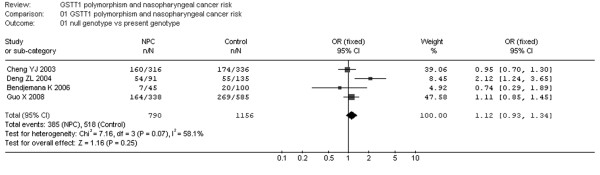
**Meta-analysis with a fixed-effect model for the association of NPC risk with GSTT1 polymorphism (null genotype versus present genotype)**.

Fig. 2 shows the association between the GSTM1 deletion and NPC risk. We analyzed the heterogeneity for all 8 studies and the test value of Chi-square was 6.73 with 7 degree of freedom (d.f.) and P > 0.05 in a fixed-effect model. For the association between the GSTT1 null genotype and NPC risk, the Chi-square value for the heterogeneity of all 4 studies was 7.16 with 3 d.f. and P > 0.05 in a fixed-effect model (Fig. 3).

**Figure 4:**
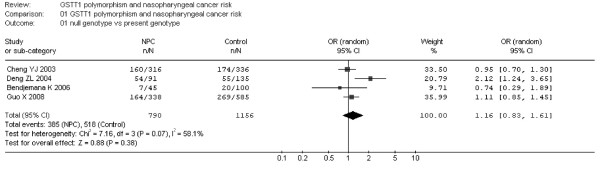
**Meta-analysis with a random-effect model for the association between NPC risk and the GSTT1 polymorphism (null genotype versus present genotype)**.

Additionally, I-square value is another index for the heterogeneity test [15], with value less than 25% indicating low, 25% to 50% indicating moderate, and greater than 50% indicating high heterogeneity. In Fig. 2, the I-square value was 0%, suggesting an absence of heterogeneity. Thus, a fixed-effect model was used. However, in Fig. 3, the I-square value was 58.1%, suggesting a possible presence of heterogeneity. Accordingly, both fixed-effect model (Fig. 3) and random-effect model (Fig. 4) were utilized for evaluation of GSTT1.

### Quantitative data synthesis

For GSTM1 polymorphism, the data available for our meta-analysis were obtained from 8 case-control studies of 1112 cases and 1601 controls, of which 649 cases and 798 controls had the null genotypes (the exposure group) and 463 cases and 803 controls had the GSTM1 present genotype. The overall OR was 1.42 (95% CI = 1.21–1.66) and the test for overall effect Z value was 4.39 (P < 0.05). The results indicate that GSTM1 null genotype might have an association with increased risks of NPC.

For GSTT1 polymorphism, the data available for our meta-analysis were obtained from 4 case-control studies of 790 cases and 1156 controls, of which 385 cases and 518 controls had the null genotypes (the exposure group) and 405 cases and 638 controls had the present genotype of the GSTT1 gene. As shown in Fig. 3, the overall OR for the null genotype versus present genotypes was 1.12 (95% CI = 0.93–1.34) and the test for overall effect Z value was 1.16 (P > 0.05) in a fixed-effect model. Moreover, the overall OR was 1.16 (95% CI = 0.83–1.61) and the Z value was 0.88 (P > 0.05) in a random-effect model (Fig. 4). Both the two models suggest that GSTT1 polymorphism is unlikely to associate with increased susceptibilities to NPC.

**Figure 5:**
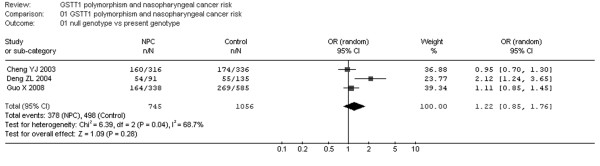
**Meta-analysis with a random-effect model for the association between NPC risk and the GSTT1 polymorphism (null genotype versus present genotype, with the reference 13 exclusion)**.

Considering that the study [13] concerning Caucasians in which the data might be different from the remaining three studies regarding Asians, we excluded it and further conducted a meta-analysis. As shown in Fig. 5, the overall OR was 1.22 (95% CI = 0.85–1.76) and the test for overall effect Z value was 1.09 (P > 0.05) in a random-effect model. Likewise, the data failed to suggest a significant association of GSTT1 deletion with NPC risk. Interestingly, the three remaining studies were conducted in China, suggesting that GSTT1 null genotype might not be the factor increasing NPC risk in Chinese population.

### Sensitivity analysis

In order to compare the difference and evaluate the sensitivity of the meta-analyses, we also reported the results of the random-effect model for GSTM1 as follows: the combined OR and 95% CI were 1.42 (95% CI = 1.21–1.66), similar to the results of the fixed-effect models. For GSTT1, the results of the fixed-effect model and random effect model were statistically similar, as stated in the above section.

Additionally, we also conducted one-way sensitivity analysis [16] to evaluate the stability of the meta-analysis. For GSTM1, the I-square value ranged from 0% to 10.4% when any single study was omitted, with the statistical significance of the overall effect size unchanged. Nevertheless, for GSTT1, the I-square value varies between 64.4% and 72% when any single study of Bendjemana [13], Cheng [11] and Guo [14] was omitted, suggesting a possible presence of heterogeneity. Notably, when the study of Deng [12] was excluded, the I-square equaled to 0%, indicating that this study [12] may contribute to the possible heterogeneity. However, the statistical significance of the overall effect size was not altered whether the study [12] was excluded or not. Hence, we could conclude that the results of the studies concerning both GSTM1 and GSTT1 are stable and credible.

#### Bias diagnostics

Funnel plots were usually created to assess the possible publication biases. In the meta-analyses, for GSTT1 and GSTM1 polymorphisms, the funnel plots were not created because it is useless when the number of the included studies is limited. Nevertheless, fail-safe number, for the evaluation of the reliability of meta-analysis, is defined as the number of negative results that could reverse the significant finding. The Nfs0.05 for GSTM1 polymorphism was 66, suggesting that the publication biases might not have a remarkable influence on the results of the meta-analyses. Notably, for GSTT1 polymorphism, it is useless to utilize fail-safe number for evaluation of publication bias when the number of the included studies is only four.

## Discussion

Previous evidence suggests that GSTM1 and GSTT1 polymorphisms may have a close association with increased susceptibility to various carcinomas. In the present study, the results of meta-analyses suggest that genetic deletion of GSTM1 may contribute to increased susceptibility to NPC whereas GSTT1 polymorphism may not.

Null mutations of GSTM1, one of the most important phase II enzymes, are known to abolish enzyme activities and therefore have been linked with increasing incidence of certain cancers, most likely due to increased susceptibilities to environmental toxins and carcinogens. Previous meta-analyses indicate that GSTM1 deficiency might have a significant association with increased risks of breast cancer [17] and lung cancer in Chinese people [18]. Our previous meta-analyses concerning oral cancer suggest that GSTM1 null genotype increases the oral cancer risk in Asians but not Caucasians [19]. However, a number of meta-analyses suggest no marked associations of GSTM1 null mutations with hepatocellular carcinoma [20], brain tumors [21], gastric cancer [22], esophageal cancer [23] and prostate cancer [24]. In this study, the results supported the notion that GSTM1 deficiency might increase susceptibility to NPC.

Similarly, null genotype of GSTT1 has been suggested to associate with risks of a number of cancers. Previous meta-analyses suggest marked associations of GSTT1 deletion with lung cancer [25], gastric cancer in Caucasians [26], brain cancers [21], colorectal cancer [27], leukaemia [28] and head and neck cancers that combined oral and pharyngeal as well as laryngeal cancers [29]. In the present meta-analysis, GSTT1 deficiency is unlikely to act as a risk factor for NPC, in line with previous meta-analyses concerning esophageal cancer [23], prostate cancer [24] and breast cancer [30], respectively. Notably, for GSTT1, the results should be interpreted with caution because of the limited number of the included studies. Moreover, for evaluation of the possible heterogeneity between the studies, we reported that the chi-square value was 7.16 and its P value was 0.07 with an I-square 58.1%, suggesting that a random-effect model should be used to address this issue. Thus, a random-effect model was used (Fig. 4). The data indicated the similarity between the two models, confirming the stability of the results.

In this meta-analysis, we did not perform subgroup analyses. First, for GSTM1 polymorphisms, the data showed an absence of heterogeneity between the included studies. In addition, the extracted data showed that most studies were conducted on Asians. Of the eight studies, only a French study concerned Caucasian while another American study concerned a combined population with several ethnicities. Hence, a subgroup analysis regarding ethnic stratification had not been performed. Second, for GSTT1 deletion, we excluded the French study that might be different from the other three studies. As a consequence, the data failed to show a significant association of GSTT1 null genotype with NPC risk in Asians (Fig. 5) or in the combined population (Fig. 3). Third, we tried to extract any data that concerned the possible relationship between smoking and alcohol addiction as well as EBV infection. Nevertheless, the primary studies did not show enough relevant information. For the same reason, the combined effects of both GSTM1 and GSTT1 deletion on NPC were not assessed. However, in the present study, we successfully extracted the necessary data from the available published papers for determination of the possible associations between these genes and NPC risk.

The results of the present meta-analysis indicated a possible role of GSTM1 deletion in the tumorigenesis and progression of NPC. Nevertheless, the data failed to show a significant association of GSTT1 null genotype with increased susceptibility to NPC. This discrepancy might be due to some reasons. For GSTT1, a gene that is highly conserved during evolution, major ethnic differences exist in frequency distribution. In East Asia, highest percentages of individuals with the GSTT1 null genotype were reported [31]. Interestingly, this incidence of NPC is high in East Asia but is low in other regions worldwide. It seems that GSTT1 deletion might have an association with increased NPC risk. Nevertheless, conversely, it indicates that although many people in East Asia carry GSTT1 null genotype and, however, only a small group of people develop NPC, implying that GSTT1 deletion might not be a key event increasing susceptibility to NPC. For GSTM1, a GST isoenzyme, has been reported to detoxify the bioreactive diol-exoxides of PAHs which is important in environmental and occupational carcinogenesis [31]. Therefore, deletion of GSTM1 might contribute to the tumorigenesis and progression of NPC. In a more recent study [32], GSTM1 but not GSTT1 null genotype was indicated to associate with head and neck cancer risk, in agreement with our study. Additionally, as the number of the included studies concerning GSTT1 was only four and their sample sizes are small, it is likely that the discrepancy may be due to chance because studies with small sample size may have insufficient statistical power to detect a slight effect or may have yielded a fluctuated risk estimate. Therefore, a number of further studies with large sample sizes are needed to address this issue.

Several limitations might be included in this study. Since most of the included studies have conducted on Asians and a few on Caucasians, the results must be interpreted with caution. Further studies concerning populations in other areas such as African and American are required to diminish the ethnic variation-produced biases. Additionally, a possible publication bias might have been introduced as only published studies written in English and Chinese as well as French that could be searched from Medline database were included. Notably, we did not use the funnel plots and Egger's linear regression test [33] for assessment of any possible publication biases because of the limited number of the included studies. Moreover, many factors may affect the results the funnel plots, leading to a misunderstanding of the publication biases [34,35]. However, the fail-safe numbers failed to indicate evident publication biases. In this study, the sample sizes of several studies in the meta-analyses are rather small, and, the pooled analyses were based upon a thousand cases and a thousand controls, which are under power to give a confirmed conclusion. Only two studies include three hundred cases and rest studies included less than one hundred cases. Authors need more cautions about their results. Furthermore, the controls of several studies were hospital-based normal individuals or patients with other diseases. In addition, whether the NPC and control groups were from the same socio-economic status or the same geographic area have not been clearly stated in some of the original papers. Hence, any selection biases might exist. Therefore, a number of further investigations regarding GSTM1 and GSTT1 polymorphisms and NPC risk are required.

In conclusion, the data of the present meta-analyses indicate GSTM1 polymorphism as a risk factor for NPC and failed to show a significant association of GSTT1 polymorphism with NPC risk.

## Competing interests

The authors declare that they have no competing interests.

## Authors' contributions

XZ and LC conceived of the study, and carried out the analysis of the literatures and drafted the manuscript. ZX carried out the collection of the literatures. QL helped with the statistical analysis and manuscript drafting. XZ conceived of the study, and participated in its design and coordination and helped to draft the manuscript. All authors read and approved the final manuscript.
